# Never Surrender

**Published:** 1886-02

**Authors:** 


### NEVER SURRENDER.

It is well to remember that the disease called consumption is not always a fatal disease, that many persons have recovered when very far gone in "last stages" of this usually fatal disease, yet the change of climate, or the getting possession of some particular food, and the rejecting of old medicines has caused many who have almost given up in despair to recover. These are facts which every physicians and almost, every family have seen. In a review of the English translation of a work by the justly celebrated professor of Medical Pathology to the Faculty of Paris, M. Jaccoud, entitled Curability of Phthisis, etc., it is announced that "the curability of phthisis is now a well-established fact." This same author then continues:

"To sum up what has been stated, pulmonary phthisis is curable in all its stages. This is the prolific notion that presides over the whole history of the disease, and which should unceasingly inspire and direct all medical action The incurability proclaimed by Laennec and his immediate successors, is disproved by pathological anatomy and clinical observatien. None should, therefore, allow themselves to be influenced by such a condemnation which is but a historical souvenir. When the existence of tubercles in the lungs is recognized, it should not be inferred from that moment that he who has them is doomed to death in consequence of their presence. Should it be found that the tubercles soften and a cavern forms, it should not be believed on this account that all is lost. It has been shown that this is not the case, and the natural tendency which tubercle has to fibrous transformation, that is to recovery, should not be forgotten. Before being discouraged, the physician should search and examine incessantly whether the patient is in the requisite conditions for such favorable evolution to occur. If all hope of absolute recovery must be abandoned, a relative cure should be wrought, and every exertion be made to place the patient in such conditions that he can live notwithstanding the lesions which are now irreparable; in a word, the plan adopted should be to strive and strive always, with the unshaken confidence which may be drawn from the notion that recovery is possible. The enemy *can* be conquered. This is the idea that should engender and sustain every effort. It is certain that this conviction is the first condition of success; since it is absence of faith in the possibility of cure which prevents the adoption of all therapeutic treatment."

---

**Pasteur** has studied the pneumo enterites of pigs, which has carried off last year in the valley of the Rhone alone more than 20,000 swine. He traces the affection to microbia.

